# Challenges to the implementation of malaria policies in Malawi

**DOI:** 10.1186/s12913-019-4032-2

**Published:** 2019-03-27

**Authors:** Chikondi A. Mwendera, Christiaan de Jager, Herbert Longwe, Save Kumwenda, Charles Hongoro, Kamija Phiri, Clifford M. Mutero

**Affiliations:** 1 University of Pretoria Institute for Sustainable Malaria Control (UP ISMC), School of Health Systems and Public Health, University of Pretoria, Private Bag X363, Pretoria, 0001 South Africa; 2 ICAP at Columbia University, Mailman School of Public Health, Pretoria, South Africa; 3 Department of Environmental Health, the Polytechnic, University of Malawi, private bag 303, Blantyre, Malawi; 4 Population Health, Health Systems and Innovation, Human Sciences Research Council (HSRC), Pretoria, South Africa; 5 School of Public Health and Family Medicine, College of Medicine, University of Malawi, Blantyre, Malawi; 6 International Centre of Insect Physiology and Ecology (ICIPE), P.O. Box 30772, Nairobi, Kenya

**Keywords:** Health policy, Policy implementation, Malaria, Malawi

## Abstract

**Background:**

Despite malaria prevention initiatives, malaria remains a major health problem in Malawi, especially for pregnant mothers and children under the age of five. To reduce the malaria burden, Malawi established its first National Malaria Control Programme in 1984. Implementation of evidence-based policies contributed to malaria prevalence dropping from 43% in 2010 to 22% in 2017. In this study, we explored challenges to implementing malaria policies in Malawi from the perspective of key stakeholders in the country.

**Methods:**

In this qualitative study, we conducted in-depth interviews with 27 key informants from April to July 2015. We stopped sampling new participants when themes became saturated. Purposive and snowballing sampling techniques were used to identify key informants including malaria researchers that were policy advisors, policy makers, programme managers, and other key stakeholders. Interviews were conducted in English, recorded and transcribed, and imported into QSR Nvivo 11 for coding and analysis. Data were analysed using the qualitative content analysis approach.

**Results:**

Participants identified three main categories of challenges to the implementation of malaria policies. First structural challenges include inadequate resources, unavailability of trained staff, poor supervision and mentorship of staff, and personnel turnover in government. The second challenge is unilateral implementation of policies. The third category is the inadequately informed policy development and includes lack of platforms to engage with communities, top-down approach in policy formulation and lack of understanding of socio-cultural factors affecting policy uptake by communities.

**Conclusions:**

Policy makers should recognize that inadequate support of policy objectives leads to an implementation gap. Therefore, policy development and implementation should not be viewed as distinct, but rather as interactive processes shaping each other. Support for health policy and systems research should be mobilized to strengthen the health system. Detailed assessment of implementation challenges to specific malaria policies should also be conducted to address these challenges and support the shift from the paradigm of malaria prevention and control to elimination in Malawi.

## Background

Health policies are major public policies instituted by governments and are described by World Health Organization (WHO) as decisions, plans, and actions undertaken to achieve specific health care goals within a society. They outline priorities and expected roles of different groups, whilst building consensus and informing people [1]. Since health policies aim to achieve improved health for the population, policies should be developed using high-quality evidence. Thus, health research has a critical role in providing evidence and reinforcing the current movement of promoting evidence-based policies [2, 3].

Whilst developing good evidenced-based policies is an initial step towards the goal of improving public health, subsequently policies need to be supported during implementation. Policy implementation is ‘what happens between policy expectations and policy results’ [4]. Since most policies stipulate ideal outcomes or intentions, inadequate policy implementation creates gaps, as are frequently observed in developing countries [5]. Challenges to implementation can be attributed to weaker health systems, which will only be strengthened if better policies are formulated and implemented [6]. Van Horn [7] proposed a conceptual model for understanding the variables shaping the link between policy and practice, which include policy resources, policy standards, communication, characteristics of implementing agencies, enforcement, disposition of implementers, political environment and socio-economic conditions.

However, policies can only be successfully implemented if the roles of key actors and the influence of a country’s political context are recognized and considered. Failure to recognise key actors and political will can result in obsolete policies, which often leads to the development of new policies when it is the processes that should be improved [8]. In addition, understanding the available policy implementation options will help to make good decisions and develop better strategies for addressing implementation challenges.

In Malawi, the government, through the Ministry of Health (MOH), is responsible for health care delivery at four levels that are linked through a referral system and include: tertiary health care in central hospitals; secondary care in district hospitals; primary care in community hospitals and health centres; and community care through health surveillance assistants and maternity clinics. Primary health care is also provided in district and central hospitals to communities in their catchment area. On average, only half of Malawian communities live within a radius of five kilometres of a health facility, making it difficult for them to access health care [9]. To support the government in health care provision, the Christian Health Association of Malawi (CHAM) has established health facilities in rural areas to provide affordable health care including the free Essential Health Package (EHP). In addition, there are private hospitals, mainly in major towns, where people who can afford medical insurance can access health care [9].

Malaria is a major health problem in Malawi affecting mainly pregnant women and children below the age of five with an estimated four million cases occurring annually [10]. The first National Malaria Control Programme (NMCP) was established in 1984 and operates under the Directorate of Preventive Health in the MOH. The NMCP is responsible for developing malaria policies and strategies and provides technical leadership for the MOH with respect to malaria prevention and control [11].

The current vision of Malawi’s malaria policy is to eliminate the disease, while its mission is to reduce the burden of malaria to a level where it is of no public health significance [12]. To support this vision and mission, specific policies and guidelines were developed in line with the WHO, Roll Back Malaria Partnership (RBM), and regional policies that account for local epidemiology and include: the National Malaria Monitoring and Evaluation Plan 2007–2011 (2007); National Malaria Treatment Guidelines (2007); National Malaria Communication Strategy (2009); Guidelines for Indoor Residual Spraying (2008); Trainers Manual on Case Management (2007); Guidelines for the Management of Long-Lasting Insecticidal Nets (LLINs) Program (2007); Guidelines for Health Surveillance Assistants for Delivery of Sulfadoxine Pyrimethamine for Intermittent Preventive Treatment (2006); Malawi Health Policy (under review); guidelines on pharmacovigilance (under development); and Guidelines for malaria Rapid Diagnostic Testing (under development) [12].

The implementation of these policies and guidelines is associated with malaria prevalence dropping from 43% in 2010 to 22% in 2017. However, malaria remains a health problem in the country [10, 13]. Two case studies have established that tangible local evidence has influenced policy development in Malawi [14, 15]. These case studies demonstrate that sound evidence-based policies have been developed in Malawi and require comprehensive support during implementation. It is thus, critical to address challenges to implementing policies, if Malawi is to shift from the paradigm of malaria prevention and control to malaria elimination as stipulated in its malaria policy [12, 16]. This study aimed to identify implementation challenges of malaria policies in Malawi from the perspective of key stakeholders.

## Methods

### Design, setting, and participants

In this qualitative study, we conducted in-depth interviews with key informants (KIs) during the period of April to July 2015. The population targeted for this study comprised policy makers; programme managers; malaria researchers, who were also policy advisors, from various research and academic institutions; and all relevant stakeholders. Participants were identified using purposive and snowballing techniques (Table 1). The number of participants in each category was determined when no new information arose during the fourth and fifth interviews. The Interviewer asked each question in different ways and at least three times until the respondents started repeating themselves. This ensured that data reached thematic saturation. The interviews lasted between 45 and 60 min and were conducted privately in the office spaces of the participants or in an environment where there were no other people present. Interviews were conducted in the three cities of Blantyre, Lilongwe and Zomba where most participants work.

**Table 1:** Details of key informants (KI) who participated in in-depth interviews to identify challenges to the implementation of malaria policies in Malawi

KI	Professional background	Experience	Role
1	Malaria Epidemiologist	11–15 years	Researcher and Policy advisor
2	Medical Epidemiologist	20–25 years	Researcher and Policy advisor
3	Medical Epidemiologist	11–15 years	Researcher and Policy advisor
4	Medical Epidemiologist	40–50 years	Researcher and Policy advisor
5	Pediatrician	30–40 years	Researcher and Policy advisor
6	Pediatrician	40–50 years	Researcher and Policy advisor
7	Medical Epidemiologist	6–10 years	Programme Manager
8	Public Health	1–5 years	Programme Manager
9	Entomologist	11–15 years	Programme Manager
10	Public Health	6–10 years	Programme Manager
11	Malaria Epidemiologist	1–5 years	Programme Manager
12	Health Economist	1–5 years	Programme Manager
13	Public Health	1–5 years	Policy maker
14	Public Health	6–10 years	Policy maker
15	Health Economist	11–15 years	Policy maker
16	Public Health	11–15 years	Policy maker
17	Health Policy Analyst	1–5 years	Policy maker
18	Public Health	6–10 years	Policy maker
19	Public Health	1–5 years	Policy maker
20	Public Health	1–5 years	Policy maker
21	Public Health	11–15 years	Stakeholder
22	Health Economist	1–5 years	Stakeholder
23	Health economist	1–5 years	Stakeholder
24	Malaria Epidemiologist	1–5 years	Stakeholder
25	Health Policy Analyst	1–5 years	Stakeholder
26	Public Health	16–20 years	Stakeholder
27	Health Policy Analyst	1–5 years	Stakeholder

### Data collection and analysis

CAM, the first author of this study was at the time of the study a PhD student, who conceived and developed the research protocol, conducted all the interviews based on the research conceptual framework. CAM is an experienced qualitative researcher who received training in qualitative research methods during his Master of Public Health (MPH) and PhD studies.

Interviews were guided by a tool with open-ended questions that were piloted prior to data collection. The questions focussed on exploring the main challenges to implementation of malaria policies developed to reduce the malaria burden in Malawi. The main question posed was *“What do you think are the barriers of implementation to sound malaria policies in Malawi?”*

English was the preferred language for all participants and the interviews were recorded using digital audio recorders. Two research assistants transcribed the recordings, which were then confirmed independently by CAM and SK for clarity and verification. The transcripts were imported into QSR Nvivo 11, a software package for organizing, managing, coding, and analysing qualitative data. The data were coded independently by CAM and SK who later compared the codes for similarity. Where differences existed, both coders revisited the transcripts together, and discussed the themes until consensus on the codes was reached. Data were analysed using qualitative content analysis principles, where data were systematically categorized using both deductive and inductive approaches [17]. Verbatim quotes have been used to illustrate points of view or concepts.

### Ethical consideration

The National Health Sciences Research Committee (NHSRC) (NHSR #1203) in Malawi and the Faculty of Health Sciences Research Ethics Committee at the University of Pretoria (Ref No. 146/2013) approved the study. All participants provided written consent before the interviews and being recorded.

## Results

In total, we interviewed 27 participants that included researchers/policy advisors (*n* = 6), policy makers (*n* = 8), programme managers (n = 6) and stakeholders (*n* = 7). The details of their professional background and years of experience are presented in Table 1.

The list of challenges ascertained from the interviews, which are presented in Table 2, have been grouped as follows: 1) structural challenges emanating from the MOH including limited commitment of both human and non-human resources for policy implementation, unavailability of trained staff, poor supervision and mentorship, and personnel turnover in government; 2) unilateral implementation of policies; and 3) inadequately informed policy development including the lack of platforms to engage with communities, top-down approach in policy development, and the lack of understanding of socio-cultural factors affecting the uptake of policies by communities.

**Table 2:** Challenges to the implementation of malaria policies in Malawi

Challenges to implementation	
a. Structural challenges	
● Inadequate resources for policy implementation	
● Unavailability of trained staff	
● Poor supervision and mentorship	
● Personnel turnover in government	
b. Unilateral implementation of policies	
c. Inadequately informed policy development	
● Lack of platforms to engage with communities	
● Top-down approach in policy development	
● Lack of understanding of socio-cultural factors affecting policy uptake by communities	

### Structural challenges

Most participants acknowledged that the government through the MOH is key in driving the implementation of health policies. Most participants, who were researchers/policy advisors, and stakeholders, strongly felt that the government needs to commit adequate resources for policy implementation. Participants felt that certain effective interventions have not been scaled up due to unavailability of resources. For example, it was noted that for greater impact, vector control interventions need high coverage in communities. Participants cited Indoor Residual Spraying (IRS), which has not been scaled up due to a shortage of resources despite being an effective and critical intervention in reducing mosquito populations. This was highlighted below:

*“IRS is still non-existent here in this country except maybe in one or two districts and even when we do it, we do it so badly”. (Researcher/policy advisor)*

Many participants identified infrastructure, such as health facilities as critical in supporting policy implementation. However, many communities in Malawi have a shortage of health facilities which means that people must travel long distances to access health care. In Malawi, the current malaria treatment policy stipulates that positive malaria cases, confirmed through microscopy or malaria Rapid Diagnostic Tests (mRDTs), should be treated within 24 h. Living far from a health facility, means that individuals coming for treatment but are misdiagnosed do not necessarily return if malaria fever resurfaces at home leading to self-treatment or complicated malaria. This concern was raised as follows:

*“The challenge is that for most people in our environment [they] live far from health facilities, [and] when you misdiagnose, and they go home it is almost impossible for them to come back in the middle of the night when the disease resurfaces again. Because sometimes malaria can’t just be diagnosed easily”. (Programme manager)*

To avoid this, health workers tend to comply with the former policy of treating patients based on the clinical presentation i.e. providing malaria treatment to every person presenting with fever.

Most participants also indicated that the lack of staff in government institutions is a huge challenge to implementation of malaria policies in the country. The shortage of health workers in many health facilities results in non-professionals carrying out activities that should be performed by trained health workers leading to poor adherence to policies. The shortage of staff also contributes to incorrect recording of information in the health passport books which is later transferred into the national Health Management Information System (HMIS). Many programme and policy decisions draw evidence from this routine monitoring and evaluation data, and when data are entered incorrectly subsequent decisions are based on incorrect information:

*“The HMIS is the main source of data to inform utilization of health services in Malawi and as much as there is now [an] improvement in reporting but the quality is not that good because the data entry in most facilities is entered by people who are just mere clerks or mere agencies who have never had a good training or understanding and have no clue on what they are doing and that data end up at very high levels so when you do validity checks you find that there are so many discrepancies between what is from the source and the reported”. (Stakeholder)*

*“sometimes the issue of short cuts is because that they are understaffed and sometimes when you visit the antenatal clinic what you find is that it is not a midwife who is completing that book, it is actually a cleaner who is completing that book and we might look very good at the books that everything is working but when you are on the ground that’s when you still see [that] the burden of malaria is still there”. (Researcher/policy Advisor)*

Some participants also identified that the shortage staff at the NMCP is affecting its operational capabilities:

*“The NMCP is doing a great job towards addressing the malaria burden in the country, however, the team at this level is small for the delivery of services. They are hence overloaded with tasks posing a challenge of not doing a thorough job on important activities … ..strengthening management and implementation will help to improve your progress”. (Stakeholder)*

In addition, the shortage of trained health workers is due to the orientation or introduction approach of new policies. The common cascade orientation approach involves the training of line supervisors who are required to train other health workers. In some cases, a top-level manager can decide to attend the training themselves, instead of assigning the responsible line supervisor, because they want to get an allowance for attendance and later provide the training documents to operational staff. In this regard, health workers who carry out routine activities, are demotivated and not trained to follow new policies. This was supported by the sentiments below:

*“If you have a new policy, I mean people [management], will just send for instance a memo during Monday morning meetings, staff meetings, whatever clinical meetings. If you send out a memo to say for instance starting from today, you need to be doing this; people will not adhere to it. They would rather want you to come, organize a work shop, and they get an allowance”. (Stakeholder)*

Participants also indicated that this challenge is exacerbated by poor supervision and mentorship once the policies have been introduced as narrated below:

*“The capacity of health workers for example must be built and after building the capacity there has to be consistent supervision and mentor them so that we sustain their capability of conducting their activities according to policy...so sometimes it’s not the case because there is no consistence so that maybe compromises in terms of malaria burden”. (Researcher/Policy Advisor)*

A few participants identified that the implementation of policies is affected by personnel turnover in government or job rotations in critical positions within the MOH. Participants mentioned that the continuity of policies becomes affected when a new administration or office bearer is reluctant to carry on the activities of the previous administration or change how the policies were implemented. This lack of continuity will always have a bearing on the impact of policies as alluded to by a policy maker:

*“But also, sometimes in our case there are certain changes that may happen for instance change of government along the way. So, when a government changes while implementing a particular policy, they say all those old policies have gone with that particular administration [and] we are new people [so] we are going in a different direction”. (Policy maker)*

### Unilateral implementation of interventions

Almost all the programme managers and some stakeholders raised the concern of unilateral implementation of interventions, which results in resource wastage and duplication of activities. Although it needs strong political commitment, integrated implementation of interventions can be very effective in reducing the malaria burden in the country. A programme manager attested to this:

*“The main prevention strategies adopted in Malawi include Insectcide Treated Nets, IPTp-SP, and IRS, so all those 3 have been tried separately rather than in combination but of course it’s quite costly”. (Programme manager).*

### Inadequately informed policy development

Most respondents agreed that policies should be developed whilst incorporating the views of relevant stakeholders such as the public. However, incorporating the views of the public is not always possible because there are no platforms for researchers or policy makers to engage with local communities, community-based organizations, and influential people including chiefs. Therefore, policies are often developed top-down, as stipulated below:

*“If you are going to really influence policy you shouldn’t only be influencing people within the ministry of health, but you should be influencing the wider community … ..I haven’t really seen that kind of a platform here in our setup which is very unfortunate because, without that platform you don’t have community based organizations, NGO's or even traditional chiefs, we don’t have influential people in the community actually contributing to the policy makeup. So, you have a bunch of people in the ministry that will decide, in my opinion that’s a lot of top down kind of approach and I don’t think that’s the way to make policy”. (Researcher/Policy advisor)*

The top-down policy development approach also affects how communities respond to the implementation of policies as they have no sense of ownership. For example, communities may accept free Long-Lasting Insecticidal Nets (LLINs) but not use them correctly. Some participants felt that policies should consider the behaviour patterns of people who will use the interventions. These concerns were raised as below;

*“We can have very beautiful policies, but we do not know the behaviour patterns of people that are being treated because we do not include them when we start looking at policies and you really do not know if those people can comply with schedules, with feeding, with taking people to the hospital. We will continue developing policies and still not solve the issue of malaria in this country”. (Programme manager)*

Participants recognized that socio-cultural factors affect policy adoption by communities, and considering these factors is very important prior to implementation. These sentiments were expressed below:

*“I think there is a huge gap already there because much as the interventions are there, I don’t think we understand very much on the cultural contexts that influence the use of some of these interventions which is quite unfortunate. Because we don’t seem to be driving a lot of research in that direction, maybe a lot of our research is a bit on the higher side where we are looking at other things rather than maybe understanding the cultural, and community issues that would affect the use of these available interventions”. (Programme manager)*

*“Qualitative issues must look at what people do when they have malaria. Focusing more on effectiveness of treatment without looking in the context at which people take medications is a big gap in malaria treatment and eradication of malaria in Malawi”. (Researcher/Policy advisor)*

Some participants acknowledged that a thorough stakeholder analysis should be conducted prior to policy development to incorporate views of relevant stakeholders who are affected or influence policy development and implementation. Thus, this approach can avoid the top-down policy development:

*“I think even policy construction is a challenge in that you may not have done thoroughly consultations and when you start implementing the policies you find that there is a lot of resistance from the ground. So, you need thorough consultations so that it’s acceptable in the society … because otherwise if you introduce something which is not acceptable then it is repelled, and it doesn’t work when you start implementing it on the ground”. (Policy maker)*

*“So, you find that if you deliberately miss out some important stakeholders then you end up developing a very beautiful policy...very colourful...but it will just gather dust in the shelf because you have not involved stakeholders”. (Stakeholder)*

## Discussion

Policies guide interventions to achieve policy objectives. However, various challenges exist during implementation despite having sound evidence-based policies [18]. It is, therefore, important to assess these challenges and devise measures of addressing them. Malaria has remained a health burden in Malawi prompting a review of the malaria policy, which strives to reduce the malaria burden to a level of no public health significance and achieve elimination [12]. This study explored challenges to implementation of the malaria policy in Malawi for policy makers to be aware of whilst developing policies.

In Malawi, the largest challenge to policy implementation, according to participants, is the lack of organisational commitment by the government. Policies can only be successfully implemented if the government shows political will by committing adequate resources for implementation. Committing resources for implementation in Malawi is incredibly difficult given the lack of both human and non-human resources. As a result, policies are often developed without setting aside sufficient resources for implementation. This form of inadequate policy development by authorities is an assumption that the policies will be implemented with minimal resources, or that policy implementation is the responsibility of other institutions [19]. Weaver [19] argued that resource availability is critical in influencing the disposition of policy implementers because inadequate resources is an obstacle. Therefore, policy makers need to consider the resources needed to implement policies whilst developing policies, thus a thorough implementation analysis needs to be conducted before embarking on policy implementation.

Human resources are vital assets for policy implementation, and the training and orientation of workers to new policies are equally important. Workers should receive clear policy communication through in-service training followed by regular supportive supervision and mentorship to ensure that they understand and comply to policy standards. In Malawi, health workers who received training and supervision were more likely to adhere to treatment guidelines in implementing the malaria “test-and-treat” policy but there was also a need for further improvement by increasing supervision and support to enforce and motivate them [20]. For example, in Tanzania continuous supervision and resource management were identified to be key for clinical staff to adhere to the new policy [21]. Poor supervision led the staff to follow their socially constructed perceptions about malaria called ‘mindlines’ which led to over-diagnosis. The three ‘mindlines’ included clinicians’ perceptions that malaria was easier to diagnose than other diseases, missing malaria was indefensible, and that malaria was a more acceptable diagnosis [21].

The shortage of health workers leads to frequent job rotations and transfers, which brings in new personnel with different levels of motivation or those who have not been trained in new policies. In this study, participants also identified personnel turnover in government to pose a challenge especially when senior position appointments within the MOH are politically motivated. The problem is further amplified when a new government administration comes into power and changes most of the technical positions [22]. New administrations may be disinclined to continue with the initiatives of the previous administration due to egotistical reasons, as was the case in Nigeria, where a previously initiated government programme was changed to suit the new administration and claim originality [5]. Leaders should always advance the needs of the people, thus, surpassing personal ambitions.

Unilateral implementation of malaria interventions has also affected the implementation of the malaria policy in Malawi. This was observed during the implementation of malaria control interventions such as LLINs, IRS, and health education. While these interventions are known to reduce malaria transmission, it is not clear if implementing them on their own can help reach malaria elimination [23]. An integrated approach to vector control has shown a remarkable reduction in the malaria burden in Zambia [24]. Through political will, the Zambian experience provides critical lessons for other malaria endemic countries such as Malawi.

The implementation of policies is also hindered by inadequately informed policy development when the views of the public, who are the primary participants of policy implementation, are not considered during policy development. As a result, communities do not take ownership of the interventions during implementation [5]. In this study, participants identified that there are no platforms where policy makers can engage with the public. Being the primary recipients of the intended and unintended effects of health policies, the public should be highly regarded during policy development, however, their involvement at this stage is often minimal [25]. Several approaches have been proposed on how to engage the public, including using journalists to engage community members through press conferences, or health articles, and tasking civil society groups with involving relevant groups of society and discussing policy issues with community members [25]. These strategies avoid the top-down approach of developing policies by incorporating the views of the public, who later feel a sense of ownership and are compelled to participate in policy implementation. In addition, health policy and systems research (HPSR) should include political, economic and socio-cultural assessments to guide both policy development and implementation, and health system strengthening [26–28]. An updated research agenda on HPSR for eliminating and eradicating malaria globally, can provide countries with a range of research questions that can be tailored to their context [29]. Furthermore, policies should be formulated with a thorough stakeholder analysis to identify community interests and perspectives that can be incorporated to enhance policy implementation [30, 31].

### Limitations of the study

Although this study is based on a small sample size, every effort was taken to reach thematic saturation. Sampling of each participant type continued until no new themes emerged.

The position of the interviewer was that of an ‘outsider’ of the government policy process and implementation, which may have limited the access and the ability to collect relevant issues pertaining to the case, and easily relate the findings to the policy environment during analysis. However, an ‘outsider’ holds an upper hand in probing for sensitive issues by being curious and make the participants to openly share their true thoughts and opinions.

The findings of this study are limited to malaria in Malawi and not necessarily generalizable to other health issues, but the approach used can inform how such studies can be conducted in similar settings. The study only included challenges to the implementation of general policies, as viewed by the selected key informants. Views of the public, who are the primary beneficiaries of health policies would have provided interesting and counterbalancing findings. Thus, further studies exploring challenges of specific malaria policies that would include views of the public are recommended.

## Conclusions

Policy makers should recognize existence of implementation gaps if policies are not fully supported. Various intervening and contextual factors will determine the direction and pace of the policy. Therefore, it is always important for policy makers not to view policy development and implementation as distinct but rather interactive processes shaping each other. Anticipating potential barriers to policy implementation during policy development allows for strategies to overcome or reduce such barriers. In Malawi, these barriers include structural challenges as identified within the government through the MOH, unilateral implementation of policies, and inadequately informed policy development.

While these factors are general challenges to the implementation of malaria policies in Malawi, they offer an opportunity for policy makers to consider issues of bridging the implementation gap during the policy development stage. Relevant stakeholders should be identified during a thorough stakeholder analysis, and policies should incorporate the views of the communities, commitment of resources and retaining competent health workers within the system, adequate monitoring of interventions, and minimizing political interference in health services.

## Abbreviations

AFIDEP: African Institute for Development Policy
COM: College of Medicine
HPSR: Health Policy and Systems Research
IPTp-SP: Intermittent Preventive Treatment with Sulfadoxine Pyrimethamine
IRS: Indoor Residual Spray
KIs: Key Informants
LLINs: Long-Lasting Insecticidal Nets
MAC: Malaria Alert Centre
MLW: Malawi-Liverpool Wellcome
MOH: Ministry of Health
NCST: National Commission for Sciences and Technology
NHSRC: National Health Sciences Research Committee
NMCP: National Malaria Control Programme
RBM: Roll Back Malaria Partnership
RDTs: Rapid Diagnostic Tests
SSDI: Support for Service Delivery Integration
USAID: United States Agency for International Development
WHO: World Health Organization
